# Ontogeny and sex alter the effect of predation on body shape in a livebearing fish: sexual dimorphism, parallelism, and costs of reproduction

**DOI:** 10.1002/ece3.278

**Published:** 2012-07

**Authors:** Elizabeth M A Hassell, Peter J Meyers, Eric J Billman, Josh E Rasmussen, Mark C Belk

**Affiliations:** 1 Department of Biology, North Carolina State UniversityRaleigh, North Carolina 27695; 2 Department of Biology, Brigham Young UniversityProvo, Utah 84602; 3 Klamath Falls Fish and Wildlife Office1936 California Avenue, Klamath Falls, Oregon 97601

**Keywords:** Age, *Brachyrhaphis rhabdophora*, environmental differences, geometric morphometrics, maturation, morphology, livebearer, physical burden

## Abstract

Predation can cause morphological divergence among populations, while ontogeny and sex often determine much of morphological diversity among individuals. We used geometric morphometrics to characterize body shape in the livebearing fish *Brachyrhaphis rhabdophora* to test for interactions between these three major shape-determining factors. We assessed shape variation between juveniles and adults of both sexes, and among adults for populations from high- and low-predation areas. Shape differed significantly between predation regimes for all juveniles regardless of sex. As males grew and matured into adults, ontogenetic shape trajectories were parallel, thus maintaining shape differences in adult males between predation environments. However, shape of adult females between predation environments followed a different pattern. As females grew and matured, ontogenetic shape trajectories converged so that shape differences were less pronounced between mature females in predator and nonpredator environments. Convergence in female body shape may indicate a trade-off between optimal shape for predator evasion versus shape required for the livebearing mode of reproduction.

## Introduction

Population divergence is an important window into evolutionary mechanisms. Local adaptation to environments with different predation regimes is one well-documented source of within-species divergence (Reznick and Endler 1982; Johnson and Belk 2001). For example, many fish species repeatedly show divergence in body shape between “predator environments,” where prey fish occur with piscivorous fish, and “nonpredator environments,” where prey fish occur without piscivores. In predator environments, these morphological differences include streamlined bodies and deeper caudal peduncles, which presumably increase escape ability and survival (Langerhans et al. 2004; Domenici et al. 2008; Gomes and Montiero 2008; Langerhans and Makowicz 2009).

While predation repeatedly generates morphological differences between populations (Langerhans 2010), other factors—such as ontogeny and sex—contribute to shape diversity among individuals (Delph 2005; Hendry et al. 2006). Ontogenetic shape change occurs as an organism increases in size and passes through functionally distinct developmental stages. (Although “ontogeny” has various meanings, here we use it to describe the transition from nonreproductive juvenile to reproductive adult.) Sexual dimorphism in shape reflects the effects of sexual selection, ecological differentiation among sexes, or the indirect effects of size dimorphism (Oliveira and Almada 1995; Fairbairn et al. 2007). We are interested in how ontogeny and sex interact with selective pressures engendered by predation to alter prey morphology. Natural selection may influence some age classes or one sex more than another (e.g., if predation mortality is higher for one sex than the other), while constraints of growth and reproduction may limit the adaptive response to predation. Either case requires an understanding of how ontogeny, sex, and predation interact to determine morphology.

Previous studies have addressed the morphological effects of ontogeny, sex, and environment, but typically not the interaction among these factors (e.g., Langerhans and DeWitt 2004; Hendry et al. 2006). If factors that influence shape act independently, then the combined effect of two or three factors will be the sum of their effects observed in isolation (i.e., additive), and the combined effect can be determined from studies addressing single factors. However, the effects of ontogeny, sex, and predation environment on shape may be interactive and thus nonadditive. They may be either subadditive, if effects from one factor oppose or constrain effects from another, or superadditive, if effects from one factor enhance effects of another (e.g., Ciannelli et al. 2004). Since body shape is an integrated, complex trait resulting from the interaction of all three factors, an integrated approach will allow us to account for potential nonadditive effects. Evaluating interactions and patterns of nonadditive effects can elucidate the path of phenotypic differentiation and help explain constraints on adaptive divergence and the mechanisms behind variation in phenotype within species.

Two specific interactions among ontogeny, sex, and environment have been of particular interest to evolutionary biologists. The first interaction addresses the question, does the (selective or plastic) effect of environmental variation on body shape constrain or magnify ontogenetic change among populations? Selection may act more strongly in one environment (Levinton 1983; Conover and Schultz 1995; Belk et al. 2005) and thus constrain or magnify patterns of ontogenetic shape change. The second interaction of interest is that between sex and environmental variation among populations. Comparing trajectories defined by this interaction addresses questions on the mechanisms and consequences of sexual dimorphism, such as, do sexes show equal magnitude and direction of divergence between contrasting selective environments? In other words, do differences in male and female reproductive roles constrain or magnify shape responses to environmental variation?

Livebearing fish of the family Poeciliidae provide ideal models for studying the interactions of multiple factors on morphology. Poeciliid fish are small bodied, mature rapidly, respond quickly to selective forces, and exist in different predation regimes (Meffe and Snelson 1989). Livebearing fish also provide good comparative data for other studies that have addressed similar questions (Langerhans et al. 2004; Gomes and Monteiro 2008).

Poeciliids are well suited for addressing our first interaction of interest (does the effect of environmental variation on body shape constrain or magnify ontogenetic change among populations?) because predation repeatedly induces significant variation among populations. Many poeciliids exhibit striking patterns of variation in life history, body shape, and coloration associated with differences in mortality caused by the presence or absence of piscine predators and accompanying environmental variables (Endler 1982; Reznick et al. 1996; Johnson and Belk 2001; Langerhans and DeWitt 2004; Hendry et al. 2006; Gomes and Monteiro 2008; Langerhans 2010). Shape divergence is particularly well documented: individuals in the presence of predators exhibit larger caudal peduncle areas and changes in the shape of the head and position of the eye (Langerhans et al. 2004; Langerhans and Makowicz 2009; Langerhans 2009a). However, how these morphological differences develop over an ontogenetic trajectory is not well studied (but see Langerhans et al. 2004).

Poeciliids are also suitable for addressing our second interaction of interest (do sexes show equal magnitude and direction of divergence between contrasting selective environments?) because most species show sexual dimorphism. Males tend to be smaller than females (Bisazza and Pilastro 1997), and sex is typically the dominant component of shape variation within a species (e.g., Hendry et al. 2006). Although many poeciliids exhibit population variation in areas of contrasting predation intensity, few studies have addressed the interaction between predation and sexual dimorphism.

We used *Brachyrhaphis rhabdophora*, a well-studied poeciliid abundant in northwestern Costa Rica, as a model to test for patterns of body shape in response to the two interactions discussed above. *Brachyrhaphis rhabdophora* exhibits strong sexual dimorphism and is found in both predator and nonpredator environments. Effects of predation regime on body shape have been quantified in adult males (Langerhans and DeWitt 2004), and predation is a key factor in the evolution of these fish (Johnson 2001a, b, 2002; Johnson and Belk 2001; Johnson and Zúñiga-Vega 2009). To test for interacting effects of ontogeny, sex, and predation environment on patterns of body shape divergence, we compared the morphology of wild-captured *B. rhabdophora* from three predator and three nonpredator locations using geometric morphometrics. We used phenotypic change vector analysis (Adams and Collyer 2007; Collyer and Adams 2007) to test how ontogeny and environment (ontogenetic trajectories) and sex and environment (sexual dimorphism trajectories) interact to determine shape variation. We predicted that the interaction between sex and environment would be nonadditive because sexes have different reproductive roles and body sizes, and thus may experience different levels of selection pressure from predators (Johnson and Zúñiga-Vega 2009).

## Methods

**Figure 1:**
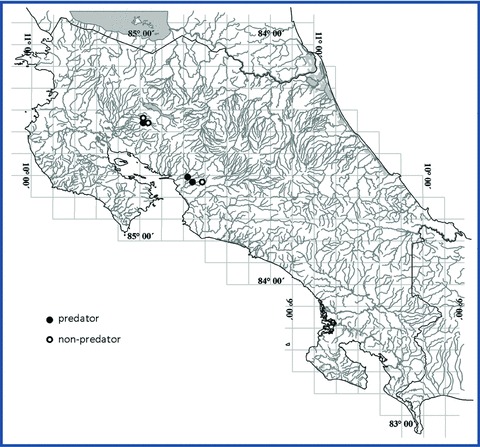
Collection sites in Costa Rica for predator and nonpredator populations of *Brachyrhaphis rhabdophora.*

Fish were collected from six streams in the Guanacaste region of Costa Rica in May 2006 (Fig. 1). In three locations, *B. rhabdophora* occurred alone or with only nonpredator species. In the other three locations, the piscivorous predators *Parachromis dovii* and occasionally *Rhamdia guatamalensis* were present. In all locations, *B. rhabdophora* occupies similar, low-velocity habitats.

We classified females as juvenile or adult based on dissections. We determined maturity in males based on the appearance of the gonopodium (i.e., mature = fully developed gonopodium; Turner 1941). A total of 676 fish comprised the study, including 360 fish from nonpredator locations (*n* = 61, *n* = 167, *n* = 132 individuals) and 316 from predator locations (*n* = 53, *n* = 211, *n* = 52 individuals).

**Figure 2:**
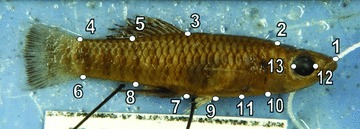
Landmarks used for shape analysis on *Brachyrhaphis rhabdophora*.

Morphometric information was acquired from a lateral image of the right side of each fish. No fish were dissected prior to being photographed. We used tpsDig2 software (Rohlf 2005) to digitize 13 biologically homologous landmarks on images (Fig. 2): (1) anterior tip of the snout, (2) posterior extent of head on dorsal outline, (3) anterior origin of the dorsal fin, (4) dorsal origin of the caudal fin, (5) semilandmark midway between landmarks 3 and 4, (6) ventral origin of the caudal fin, (7) anterior origin of the anal fin (gonopodium for males), (8) semilandmark midway between landmarks 6 and 7, (9) anterior insertion of the pelvic fin, (10) intersection of the operculum with the ventral outline of the body, (11) semilandmark midway between landmarks 9 and 10, (12) most anterior point of eye outline, and (13) most posterior point of eye outline. Semilandmarks are considered mathematically fixed in one direction and only vary along the axis perpendicular to the line connecting the two points between which the semilandmark is placed. We marked semilandmarks by visually approximating the half-way points between landmarks on either side. Three additional points placed along the lateral midline were used to facilitate digital unbending of the specimens in tpsUtil (Rohlf 2004), but were not used in the final analyses.

We used digitized landmarks to obtain shape variables in tpsRelw (Rohlf 2003b) for analyses. First, generalized Procrustes analysis (Rohlf and Slice 1990) was used to remove all nonshape variation due to position, orientation, and scale of the specimen in each image. The resulting data were superimposed to generate aligned specimens that were used to calculate affine and nonaffine shape components (i.e., uniform components and partial warps). Relative warp scores (i.e., principal component scores) were calculated from a principal components analysis of the partial warps and uniform components and were used as shape variables (response variable) in statistical analyses. We used the first 12 relative warps in statistical analyses to account for the majority of the shape variation (96%) while still reducing the number of warps to account for the univariate nature of semilandmarks.

We analyzed shape variation with a multivariate mixed model using proc MIXED in SAS (2008). This procedure is an area of relatively new development and has the advantage of allowing us to handle multiple populations as random factors (mixed model) while incorporating all shape variables simultaneously (multivariate analysis). Response variables were shape characterized as relative warp scores for the first 12 relative warps. Because digitized landmarks, and therefore relative warps, are treated as repeated measures on individuals, individual and locations within predation regime were considered random variables in each model. Centroid size is often used as a covariate in morphometric analyses to account for possible allometric effects of size that are not accounted for by the scaling function of superimposition. While often a useful and necessary approach, under some conditions including centroid size as a covariate is not appropriate. We chose not to include centroid size as a covariate in this analysis for two reasons. First, use of a covariate is only valid when the range of values is common to all treatment combinations. Some of our treatment groups exhibited nonoverlapping size distributions (e.g., adults vs. juveniles). Second, size differences are already included in the model in the form of our three main effects (sex, ontogenetic stage, and predation environment). Since ontogenetic stage effectively substitutes for size, and since we do not want to test size specifically, including centroid size in this model would be redundant and would not allow a test of the focal hypotheses.

*Brachyrhaphis rhabdophora* are sexually dimorphic; therefore, we conducted separate analyses on each sex to determine how the main effects of predation regime (predator or nonpredator) and ontogenetic stage (designated as juvenile or adult) interacted to cause morphological divergence within each sex. Additionally, we conducted an analysis with only adult individuals with predation regime and sex as main effects to determine how predation affects sexual dimorphism in this species. All interactions among main effects were included in each analysis. We used the Kenward–Rogers method for estimating degrees of freedom for all terms in the mixed model (SAS 2008).

Analysis of multivariate response variables (i.e., shape variables in this case) in a mixed model requires the multivariate data to be treated as a repeated measure. Because relative warps are ordered variables, an index variable that represents the order of relative warps must be included as a fixed categorical effect in the model, similar to a time variable that represents the order of sampling events in a repeated measures analysis (Wesner et al. 2011). The model also includes interactions of the other fixed effects with the index variable. Thus, main effects by themselves test the hypothesis that shape varies between levels of the main effect on average across all relative warps, whereas the main effect by index variable interaction tests the hypothesis that shape varies between levels of the main effect on at least some warps. Because relative warps are principle components, they are orthogonal to each other and the direction and magnitude of differences between levels of main effects on one warp has no bearing on the differences (direction or magnitude) between levels on other relative warps. Because of this characteristic of relative warps it is unlikely that a main effect by itself would be significant even if shape differed dramatically between levels of the main effect, because the differences between levels of the main effect would be unlikely to correspond in direction, by chance, across most relative warps. However, the main effect by index variable interaction would be significant as long as the differences between levels of the main effect were observed on at least some warps irrespective of the direction of difference (Rencher 2002; Butler et al. 2007). Thus, it is the interaction between main effects and the index variable that tests the hypothesis of interest in this type of model.

We used phenotypic change vector analysis (PCVA; Adams and Collyer 2007; Collyer and Adams 2007) to test for the interacting effects of ontogeny and predation regime and of sex and predation regime on morphological divergence. A significant interaction term between main effects in the MANOVA indicates that the pattern of phenotypic change is different; however, it is difficult to determine if the differences are caused by the magnitude or direction of change. PCVA was used to compare ontogenetic shape trajectories for each sex to further examine significant interactions between predation regime, ontogenetic stage, and the index variable. We compared attributes of the phenotypic trajectories (i.e., size and direction, or angle between trajectories) to determine how phenotypic change across ontogeny differs between predation regimes for each sex. A significant difference in trajectory length represents a difference in the magnitude of morphological change, while a significant angle between trajectories represents a divergence or convergence in shape. The PCVA was also used to examine a significant interaction between predation regime, sex, and the index variable to compare the amount and direction of sexual dimorphism between predation regimes. In this analysis, a significant difference in trajectory length between sexes represents differences in the magnitude of dimorphism, while a significant difference in direction of change would indicate that predation affects males and females differently. The phenotypic trajectory analyses were conducted using ASREML-R version 2.00 (Butler et al. 2007) within R (R v.2.8.1; R Development Core Team 2008).

To visualize differences in shape for adults and juveniles of each sex, we derived a canonical axis from the predator regime factor effect (i.e., fish from predator environments on one end of the axis and fish from nonpredator environments on the other) using methods described by Langerhans et al. (2004). Briefly, we examined correlations between superimposed landmark coordinates and the predation regime axis generated using proc CANCOR in SAS (2008). For visualization, thin-plate spline transformations were generated in tpsRegr (Rohlf 2003a) using canonical scores and superimposed landmark coordinates.

## Results

**Figure 3:**
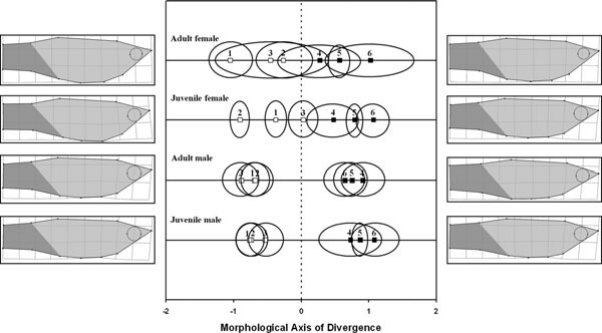
Morphological axes of divergence of *Brachyrhaphis rhabdophora* based on canonical variants derived from the predation environment factor based on predation regime (filled symbols = predator environments; open symbols = nonpredator environments). Ellipses represent 95% confidence regions along the axis for each population. Canonical correlations were conducted separately for each gender and age combination (seven separate axes presented). Thin-plate spline transformation grids are provided at the ends of each axis; grids represent axes’ endpoints to better illustrate morphological differences.

**Figure 4:**
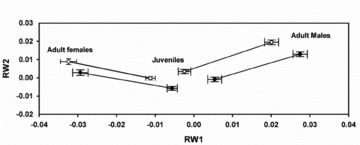
Relative warp plot of least squares means (±1 SE) for juvenile and adult male and female *Brachyrhaphis rhabdophora* from predator (filled symbols) and nonpredator (open symbols) environments. The first two relative warps account for 34.5% and 17% of the variation, respectively.

In the comparison of female shape, both the interaction of predation regime by index variable and the interaction of ontogenetic stage by index variable were significant (Table 1). The interaction between predation regime, ontogenetic stage, and the index variable was marginally significant (*P* = 0.073), so the trajectories were compared with the phenotypic change vector analysis. The lengths of the vectors were not significantly different (lengths = 0.027 and 0.026, |d1 – d2| = 0.001, *P*_rand_ = 0.920), indicating that the amount of phenotypic change through ontogeny was the same regardless of predation regime. However, the angle between vectors (21.15°) was significant (*P*_rand_ = 0.025). This represents convergence of shape in adult females (Figs. 3 and 4; this is less obvious from Fig. 4, a simplified representation of an analysis with multiple dimensions). Juvenile females in predator locations had a larger caudal peduncle, a more elongate body, a more anterior placement of the eye, and a shorter and less up-turned head, but these shape differences were reduced in adults (Fig. 3).

**Table 1:** Results of mixed repeated measures MANOVA examining shape variation in *Brachyrhaphis rhabdophora*. Shape variation of individual sexes was examined to determine the effect of predation on ontogenetic changes in morphology. Shape variation of adult females and males was examined to determine the effect of predation on sexual dimorphism

	Effect	Degrees of freedom	*F*	*P*
Females				
	Predation	1/7.45	2.82	0.134
	Ontogenetic stage	1/2577	36.40	<0.001
	Index variable	11/1694	57.06	<0.001
	Predation × index variable	11/1694	11.73	<0.001
	Ontogenetic stage × index variable	11/1694	31.22	<0.001
	Predation × ontogenetic stage × index variable	12/1683	1.65	0.073
Males				
	Predation	1/5.68	0.08	0.785
	Ontogenetic stage	1/2189	143.36	<0.001
	Index variable	11/1409	40.63	<0.001
	Predation × index variable	11/1409	16.16	<0.001
	Ontogenetic stage × index variable	11/1409	40.16	<0.001
	Predation × ontogenetic stage × index variable	12/1404	1.47	0.128
Adults				
	Predation	1/6.48	1.18	0.316
	Sex	1/1410	484.75	<0.001
	Index variable	11/1111	36.62	<0.001
	Predation × index variable	11/1111	8.53	<0.001
	Sex × index variable	11/1111	69.67	<0.001
	Predation × sex × index variable	12/1110	2.82	0.001

Male shape differed significantly by the interactions of predation regime by index variable and ontogenetic stage by index variable, but the interaction between predation regime, ontogenetic stage, and the index variable was not significant (*P* = 0.128; Table 1). This indicates that the amount and direction of shape change across ontogeny was the same between predation regimes (Fig. 4). Males in predator locations had a more elongate body, a more anterior and dorsal placement of the eye, and a less up-turned head (Fig. 3).

Morphology of adult fish differed significantly by the interactions of predation regime by index variable and sex by index variable, and the interaction between predation regime, sex, and the index variable was also significant (*P* = 0.001; Table 1). Trajectories representing sexual dimorphism between predation regimes exhibited no difference in vector length (lengths = 0.056 and 0.058, |d1 – d2| = 0.002, *P*_rand_ = 0.673), but a significant angle between vectors (12.43°; *P*_rand_ = 0.002). The angle between the trajectories of sexual dimorphism indicates convergence in adult female morphology relative to adult males.

## Discussion

Ontogenetic stage (juvenile or adult) and sexual dimorphism accounted for the greatest amount of shape variation within *B. rhabdophora* (Table 2). In both males and females, juveniles had relatively large heads and small, streamlined bodies compared to adult forms. This pattern is consistent with ontogenetic shape change observed in many species of fish (Loy et al. 1996; Reis et al. 1998; Tomeček et al. 2005; Ristovska et al. 2008), and has been interpreted as a juvenile morphology that emphasizes early development of sensory and feeding systems (i.e., eyes and mouth size; Osse 1990; Osse et al. 1997; Gisbert 1999). Between adult males and females, the primary morphological differences lie in the shape and relative size of the abdomen. Males develop a gonopodium and its associated muscular and skeletal structure (i.e., suspensorium) in the anal area (Turner 1941; Reznick et al. 1993). Females develop an expanded abdominal area associated with pregnancy and internal development of embryos, and the volumetric constraints imposed by livebearing (Weeks 1996). Thus, the general pattern of intraspecific shape diversity is in the change from a shape that emphasizes sensory abilities and feeding in early juveniles to a shape determined by the requirements of reproduction and the different roles of males and females in a livebearing reproductive mode.

**Table 2:** Variation accounted for by relative warps used in the analysis

RW no.	Singular values	Percent explained (%)	Cumulative percent explained (%)
1	0.63474	34.48	34.48
2	0.44622	17.04	51.51
3	0.38339	12.58	64.09
4	0.33511	9.61	73.70
5	0.24688	5.22	78.92
6	0.24206	5.01	83.93
7	0.19564	3.28	87.21
8	0.16720	2.39	89.60
9	0.15532	2.06	91.66
10	0.14246	1.74	93.40
11	0.12769	1.40	94.80
12	0.10725	0.98	95.78

Although ontogeny and sex account for a large amount of variation in body morphology, predation environment is also responsible for a significant amount of variation. Fish from predator environments had narrower bodies and showed shifts in morphology similar to those documented in other poeciliids (Langerhans et al. 2004; Langerhans 2009a; Langerhans and Makowicz 2009). However, patterns of divergence through ontogeny were not the same for both sexes; the effect of predation had a significant interaction with female ontogeny (Table 1). For males, the magnitude of morphological differences resulting from predation regime was constant for both juveniles and adults. In contrast, for females, morphological differences between predation regimes changed across ontogeny: differences observed in juveniles were lessened in adults. This is indicated by the significant angle between trajectories of female ontogenetic change in predator and nonpredator environments and between females, but not males, in the sexual dimorphism phenotypic trajectory. This female convergence is possibly a consequence of the viviparous reproductive mode, since pregnancy results in similar morphologies (e.g., Plaut 2002) regardless of predation regime.

Female shape convergence brings up a possible explanation for the costs of reproduction. (By convergence we do not mean that female shape is identical between populations, but rather that differences between adult females in contrasting predator environments are *less* than the differences between juvenile females.) Reproduction—especially through live birth—has a significant price, including decreased locomotor ability in gravid animals (Magnhagen 1991) probably due to physiological changes, the addition of mass, and, in an aquatic environment, increased drag (Bauwens and Theon 1981; Miles et al. 2000; Olsson et al. 2000; Webb 2004). The mechanism behind the constraining effect of a physical burden is not well elucidated. Our results suggest that one potential explanation for reduced locomotor ability is convergence of shape as a constraint of livebearing reproduction. The fact that shape converges in adult females could mean that physical changes of pregnancy may be somewhat difficult to avoid because of inherent trade-offs (i.e., reproduction vs. escape ability; Ghalambor et al. 2004). Additionally, the exponential relationship between velocity and drag may mean that the relative cost of maintaining a predation morphology increases slightly for reproductive females since a slower moving female (due to physiological strain or shape constraints of pregnancy) would benefit considerably less from extreme streamlining than a fast-moving female.

Female shape convergence also sheds light on previously documented patterns of survival in *B. rhabdophora*. Survivorship is higher in nonpredator environments compared to predator environments. In the presence of predators, survival rates across age classes are relatively constant until the largest adult stage when survival decreases; in contrast, survival of the largest adult stage increases in nonpredator populations (Johnson and Zúñiga-Vega 2009). Pregnancy has a significant, negative effect on burst speed, and thus predator avoidance, in females of other livebearing fish (Ghalambor et al. 2004; Belk and Tuckfield 2010). Convergence in shape of females coincides with a divergence in survivorship between predator and nonpredator environments, suggesting that shape constraints as a consequence of pregnancy could be responsible for increased mortality rates in predator environments. An interesting direction for further work would be to compare male and female survival rates in light of their morphological responses to predation.

Overall, shape differences between predator and nonpredator environments likely indicate a trade-off between different types of swimming specialization. In predator environments, selection favors short bursts of speed during predator encounters (Webb 1986; Walker et al. 2005). However, in nonpredator environments, sustained swimming appropriate for constant foraging is favored (Langerhans 2009b). These two types of swimming are maximized by different body types (Webb 1982), so that the best shape for evading predators comes at the cost of foraging efficiency. For males, females, and juveniles in predator environments, we observed heritable shape phenotypes that are associated with greater burst-swimming speeds (*Gambusia affinis*; Langerhans et al. 2004). Since *B. rhabdophora* in predator environments have higher mortality (Johnson and Zúñiga-Vega 2009), a burst-swimming morphology could significantly enhance fitness in this system.

Compared to swimming differences between populations and species, less work has investigated how swimming specialization changes over a fish's lifetime. The course of ontogenetic development in this study suggests that swimming ability over ontogeny may progress from unsteady to more steady swimming ability in both predator and nonpredator environments (Fig. 4). Thus, the differences in morphology between environments may reflect the position of the population along the ontogenetic development trajectory favored by the trade-off between burst swimming and sustained swimming.

In conclusion, morphological responses to predation are visibly similar in both sexes at a young age and consistent with predictions for enhanced escape ability. Adult males retain these differences throughout life, yielding distinct, yet parallel, ontogenetic trajectories. Ontogenetic trajectories of females converge, pointing to a fitness compromise between reproduction and survival.
